# Male Endurance Athletes: Examination of Energy and Carbohydrate Availability and Hormone Responses

**DOI:** 10.3390/nu16213729

**Published:** 2024-10-31

**Authors:** Erin M. Moore, Clemens Drenowatz, Brittany T. Williams, Thaddeus C. Brodrick, David F. Stodden, Toni M. Torres-McGehee

**Affiliations:** 1 Department of Kinesiology, School of Education and Human Development, University of Virginia, Charlottesville, VA 22904, USA; 2 Department of Teacher Education, University of Education Upper Austria, 4020 Linz, Austria; clemens.drenowatz@ph-ooe.at; 3 Department of Exercise Science, Arnold School of Public Health, University of South Carolina, Columbia, SC 29208, USA; 4 Department of Educational and Developmental Science, College of Education, University of South Carolina, Columbia, SC 29208, USA; stodden@mailbox.sc.edu; 5 Office of Access and Collective Engagement, Arnold School of Public Health, University of South Carolina, Columbia, SC 29208, USA; torresmc@mailbox.sc.edu

**Keywords:** low energy availability, low carbohydrate availability, testosterone, leptin

## Abstract

Background: This study investigated the effects of decreased energy availability (EA) and carbohydrate availability (CA) on reproductive and metabolic hormones in male endurance-trained athletes. Methods: Thirteen athletes (age: 26.08 ± 4.3 years; weight: 70.9 ± 6.5 kg; height: 179.9 ± 4.2 cm) participated in two training weeks with varying training volumes (low [LV] and high [HV]). The participants logged their diet and exercise for seven days and provided blood samples to measure hormone levels (Testosterone [T], insulin, leptin, cortisol, and interleukin-6 [IL-6]). Results: Results showed that 46.2% (HV) and 38.5% (LV) of participants were at risk for low EA (≤25 kcal/kg FFM·d-1), while 53.8% (HV) and 69.2% (LV) had low CA (<6 g/kg). Strong positive correlations were found between leptin and body fat percentage (DXABFP) in both weeks (HV: r(11) = 0.88, *p* < 0.001; LV: r(11) = 0.93, *p* < 0.001). Moderate correlations were observed between T and DXABFP (r(11) = 0.56, *p* = 0.05) and negative correlations between leptin and fat intake (r(11) = −0.60, *p* = 0.03). Regression analyses indicated significant relationships between DXABFP and T (F(1,11) = 4.91, *p* = 0.049), leptin (HV: F(1,11) = 40.56, *p* < 0.001; LV: F(1,11) = 74.67, *p* < 0.001), and cortisol (F(1,11) = 6.69, *p* = 0.025). Conclusions: These findings suggest that monitoring body composition and macronutrients can be clinically useful for male athletes, especially those without access to blood testing. Ultimately, a greater understanding of health and performance outcomes for male athletes is needed.

## 1. Introduction

The increased participation in high-energy demanding exercise has changed from purely elite sports participation to recreational athletes. Endurance runners are unique athletes due to the high-energy physical demands of the sport. These increased demands escalate endurance athletes’ (i.e., distance runners, triathletes, etc.) risk of impaired physiological functions (e.g., compromised hormonal profile (Testosterone (T), leptin, insulin, cortisol), decreased bone mineral density (BMD), and compromised macronutrient profile [1,2,3,4,5,6,7,8,9,10,11,12,13]. However, it is unclear how male endurance athletes’ potential physical impairments are compared to more well-established research on female endurance athletes [1,2,3,4,8,9,10,11,12,13].

Relative Energy Deficiency in Sports (RED-S) and Male Athlete Triad (Triad) statements have sparked research in male athletes regarding negative impacts on health and performance due to low energy availability (LEA) with or without an eating disorder (ED)/disordered eating (DE) [2,3,4,11,12,13,14]. While the Male Triad focuses on bone mineral density, reproductive hormones (testosterone [T] and luteinizing hormone [LH]), and LEA with or without ED/DE, RED-S’ focus is more wide-ranging. RED-S encompasses various levels of energy intake concerns, including LEA, low carbohydrate availability (LCA), metabolic hormones (i.e., cortisol, leptin, insulin, interleukin-6 [IL-6], bone markers, etc.), various other body symptoms (neurological, cardiovascular, skeletal muscles, hematological, etc.) and performance measures (sleep, power, strength, motivation, etc.). To date, ~48% of male Olympic-level athletes presented with at least 1 RED-S symptom, while ~16% presented with two or more RED-S symptoms [13]. In males, LEA has been demonstrated in the literature to range from ~9–30 kcal/kg FFM/day [4,9,10,13,15,16,17,18]. Similar to females, LEA can suppress the hypothalamic-pituitary axis, which affects reproductive hormones T and LH, as well as secondary and tertiary effects on metabolic pathways, including cortisol, insulin, leptin, resting metabolic rates (RMR), body fat percentage, and bone health [3,4,11,12]. Existing literature has established acute and long periods of restricted EI or increased exercise energy expenditure (EEE) in male athletes and soldiers with negative reproductive and metabolic hormonal effects [5,15,19,20,21,22,23,24].

There is inconsistent knowledge within existing literature for male athletes participating in high EEE activities with decreased energy needs. Understanding the physiological demands and consequences of increased EEE, reduced energy intake (EI), compromised energy availability (EA), and carbohydrates (CHO) in male athletes is critical for acute and long-term health and preventing injuries and illness. Our study examined the effect of EA and carbohydrate availability (CA) on reproductive (T and LH) and metabolic hormones (insulin, leptin, cortisol, and IL-6) in male endurance-trained athletes. We hypothesized that male endurance-trained athletes who displayed LEA or LCA would present with decreased T, LH, insulin, and leptin and increased cortisol and IL-6. A secondary purpose aimed to examine secondary measures including EI, EEE, dual-energy X-ray absorptiometry body fat percentage (DXA BFP), resting metabolic rate (RMR), and mileage) and their relationships to the reproductive (T and LH) and metabolic hormones (insulin, leptin, cortisol, IL-6). We hypothesized that some secondary measures would demonstrate negative relationships with the hormones.

## 2. Materials and Methods

A within-subject cross-sectional design was implemented on male recreational endurance runners, including obstacle racers, triathletes, and distance runners. Data collection included EA, EEE, BMD, and the hormones T, LH, leptin, insulin, cortisol, and IL-6. This study was part of a larger project and utilized the same methodology as Moore et al., 2021 [18].

Participants: Thirteen endurance-trained male athletes (age: 25.7 ± 3.8 yrs; weight: 70.9 ± 6.9 kg; and height: 179.4 ± 4.5 cm) were recruited from the local community in the southeastern US. Specific inclusion criteria for participation included: males within a competitive season (i.e., a minimum of one race within three months during and after data collection), actively training and competing > 10 h/week for at least three months [19,25,26], a bioelectric impedance analysis (BIA) body fat percentage (BFP) ≤ 12% [5,19,25,26,27], maintained weight stability (±3 kg in past six months) [15], a VO_2_max that is considered excellent for age-specific range [28], and were required to be independent of any injury that would prevent them from full participation in their chosen high-endurance sport (running, triathlon, or obstacle racing). Specific exclusion criteria included no previous history, past or present diagnosis of clinical eating disorder, history of cardiovascular disease, thyroid, pituitary disease, or metabolic disease. Institutional Review Board approval was obtained before the start of the study, and all participants provided written consent before participation.

Basic demographic and multiple anthropometric measurements, including height, weight, and body composition, were collected according to ACSM standardized procedures [29]. A Tanita scale (Tanita Co., Tokyo, Japan) was used to preliminarily verify the inclusion criterion, and DXA (GE Healthcare Lunar Prodigy densitometer, Madison, WI, USA); used for data analysis) was used to assess body fat. Resting Metabolic Rate (RMR) was measured using indirect calorimetry (Microlife MedGem; HealthTech, Golden, CO, USA), a clinically validated measurement device that assesses RMR [30]. Predicted RMR was calculated with the Ten-Haaf equation as it predicted 80.2% ± 10% of measured values compared to other equations [31]. RMR ratios were calculated using RMR measured/RMR predicted [32]. Ratios below 0.90 were considered low [32,33].

Energy Needs: Multiple measures were collected, including RMR, EI, EEE, total daily energy expenditure (TDEE), distance (mileage), and resultant EA and energy balance (EB). Calculated individual VO_2max_–HR regression slopes were assessed and reported in kcals to calculate EEE using HR data [25]. Dietary records were analyzed for total kilocalories consumption using a dietary analysis software program (ESHA food processor 8.0, Salem, OR). A 7-consecutive day weighted diet record demonstrates superior accuracy compared to a food-frequency questionnaire [34]. Food records were used to examine EI and EA, defined as the amount of dietary energy remaining after exercise, expressed as kcal/kg/free-fat mass (EA = [EI–EEE]kcal/kgFFM·d^−1^) [3,4,11,12,13]. LEA was defined as ≤25 kcal/kgFFM·d^−1^ based on EI and EEE calculations for the two separate training weeks. LCA was defined as <6 g/kg.

Blood Samples: Fasting blood samples were acquired following 24 h of abstention from exercise at the end of both training weeks. Samples were taken from the antecubital space, centrifuged, and pipetted into 2-mL polyethylene tubes for storage in a −80 °C freezer for one month before analysis. Blood samples were assessed using enzyme-linked immunosorbent assay (ELISA) kits for six hormones (T, LH, insulin, leptin, cortisol, and IL-6). The ELISA kits were ascertained from R&D Systems (R&D Systems, Inc., Minneapolis, MN, USA) and Mercodia AB (Uppsala, Sweden) for Human IL-6 Quantikine HS Elisa Kit, Testosterone Parameter Assay Kit, Cortisol Parameter Assay kit, Human Leptin Quantikine Elisa Kit, Human C-Peptide Quantikine Elisa Kit, Human Hormone-sensitive Lipase/HSL Elisa kit, and Mercodia Insulin Elisa kit. The sensitivity of ELISAs is high, with a 1–10 ug/liter range and a reported correlation coefficient between 0.95–0.99 [35]. The establishment of cutoffs was identified as (1) low, (2) within normal limits, or (3) high based on previously established normative data specific for males (adult and age range specific). Normative ranges for each hormone include: T = 270–1070 ng/dL (average 679 ng/dL) [36], LH = 1.24–7.8 mlU/L [36,37], fasting insulin = ≤ 5 ulU/mL (8–10 ulU/mL is also an accepted range) [38], leptin = 0.5–12.5 ng/mL [39], cortisol specific to the morning includes: 7–28 ug/dL [40] and IL-6 =< 1.8 pg/mL [40].

Training Conditions: Two separate training weeks were used to assess differences between EA, CA, and hormone levels. A high-volume (HV) training week consisted of ≥5 days of training and included ≥10 h of training within a 7-consecutive day week. A low-volume (LV) training week (identified as a recovery week) was defined as an unloading week for the participant. No specific requirements were established for the LV week, except participants were asked to work out a minimum of 2–3 days for the 7-consecutive-day week.

**Figure 1:**
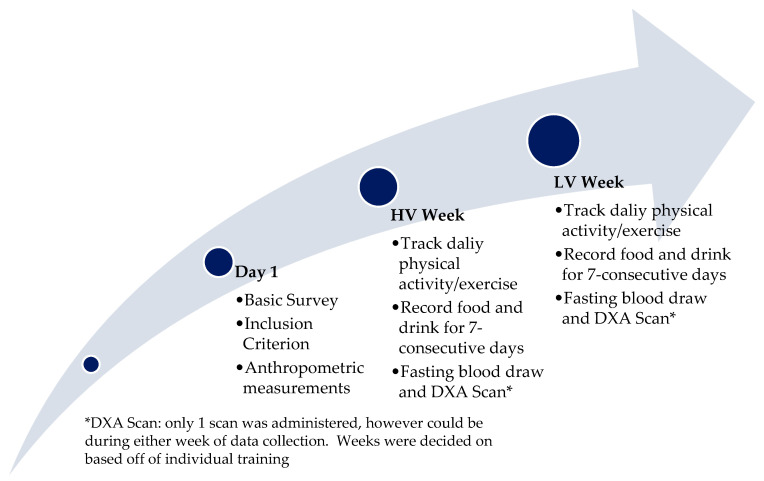
Study Procedure.

Procedures: Participants were instructed not to alter their daily/weekly activities and exercise and to record their food and daily training for seven consecutive days. They were instructed to wear an HR monitor only during exercise (to calculate EEE) during training for seven consecutive days (Figure 1).

Statistical Analysis: IBM SPSS statistical Software (version 26; SPSS Inc., Armonk, NY, USA) and an alpha error (α) of ≤ 0.05 were used for all analyses to determine statistical significance. An a priori power analysis using G*Power software (version 3.1.97, Heinrich Heine University, Dusseldorf, Germany) calculated power using chi-square analysis for LEA and LCA risk, with an alpha of 0.05. Based on previous literature by Loucks et al. [41] and Koehler et al. [15], an effect size between 1.0 and 3.0 was indicated, requiring a sample size of 2–16 subjects. Using the Wilcoxon signed-rank test with an effect size of 1.0, 13 subjects allow for full saturation, with power at 0.951 [18]. Descriptive statistics for all dependent variables were calculated with frequencies and proportions with 95% confidence intervals for all categorical variables (at risk for LEA, at risk for LCA, at risk for compromised hormonal profile), and chi-square analysis was used to examine “at risk” for LEA and LCA. Pearson’s correlations and linear regressions were used to investigate relationships and predictive qualities among EA, carbohydrate availability hormones (T, leptin, cortisol, insulin, and IL6), and secondary measures (e.g., DXA BFP, EI, EEE, CHO, RMR ratio, etc.).

## 3. Results

Eighteen participants began the study. Four were eliminated for lack of compliance with the required procedures, and one was removed as an outlier due to falling 3+ standard deviations from hormonal profiles. Thus, data from 13 participants were used for analysis. The participants’ inclusion criteria and demographic information are reported in Table 1.

### 3.1. Energy Needs

Means, standard deviations, and paired samples *t*-tests were calculated to compare the two training weeks for energy needs and hormones. There were no significant differences between the weeks except for EEE (*p* = 0.01) and TDEE (*p* = 0.03). The participants demonstrated LEA (≤25 kcal/kg FFM) during both training weeks, with 46.2% (*n* = 6) during the HV training week and 38.5% (*n* = 5) during the LV training week. When examining the ≤30 kcal/kg FFM mark, over half presented with LEA in both weeks (HV: 61.5%, *n* = 8; LV: 53.8%, *n* = 7). Most participants presented with LCA (<6 g/kg) over both training weeks. Within the HV week, 53.8% (*n* = 7) consumed less than 6 g/kg, and 69.2% (*n* = 9) under-consumed CHO (<6 g/kg) in the LV training week. Most participants had a negative energy balance for both weeks (61.5% *n* = 8). A significant moderate Pearson’s correlation was found between the HV training week volume (minutes) and CHO intake (r(11) = 0.71, *p* = 0.01). Suggesting that during the HV training week, training volume in minutes is linked to CHO intake values. A significant linear relationship was found between HV training week and CHO intake and training volume (minutes) F(1,11) = 10.84, *p* = 0.01), and produced R^2^ of 0.496. The participants’ predicted CHO intake during the HV training week was 247.16 + 0.283 (training volume minutes). The participants’ predicted CHO intake (g) in the HV training week increased by 0.283 g for each minute of training volume. The results of the participants’ energy needs with paired *t*-tests are reported in Table 2.

### 3.2. Hormones

No significant differences were found in hormone levels between the two training weeks (see Table 3). When examining mean hormone responses, participants were low in leptin and IL6. Other hormones demonstrated levels within normal ranges (cortisol for both weeks and insulin HV week), while some hormones were elevated (T and LH for both weeks and insulin LV week). The participants’ hormone profiles with paired *t*-test results are reported in Table 3. All participants elicited low IL6 levels for both weeks; 38.5% (*n* = 5) participants were low in leptin during the HV week, while 53.8% (*n* = 7) were low in leptin during the LV week. A large percentage of participants demonstrated high levels of T (>1070 ng/dL), with 61.3% (*n* = 8) in the HV training weeks and 53.8% (*n* = 7) in the LV training week.

No correlation or regression was conducted with LH due to its pulsatile nature, which, therefore, would not be a strong representation of that hormone within the analysis. When observing hormone levels in relation to LEA (≤25 kcal/kg FFM^.^ day^−1^) and LCA (<6 g/kg), IL6 and T hormones presented with compromised changes in participants, while leptin presented a significantly compromised change in relation to LCA (see Table 4), all other hormones were within normal limits.

### 3.3. Energy Needs Relationship to Hormones

Pearson correlations were calculated to examine the relationships between Hormones (T, leptin, insulin, and IL6) and multiple variables (DXA BFP, RMR ratio, caloric and macronutrient [fat kcal and CHO g/kg] intake]). Strong positive correlations were found between leptin and DXA BFP for both training weeks (HV: (r(11) = 0.88, *p* < 0.001 and LV: (r(11) = 0.93, *p* < 0.001), indicating a significant linear relationship between the two variables. Leptin in both training weeks increases with body fat percentage. The relationship between participants’ EA in the LV training week and LV CHO intake (g) displayed a strong positive correlation (r(11) = 0.81, *p* < 0.001), indicating a significant linear relationship between the two variables. Leptin levels during the LV training week and RMR ratio elicited a moderate negative correlation (r(11) = 0.63, *p* = 0.02). This indicates that leptin levels during the LV training week are linked to a lower RMR ratio. EA in the LV training week increases with carbohydrate intake. A moderate positive correlation was found between participants’ HV T and DXA BFP (r(11) = 0.56, *p* = 0.05), indicating a significant linear relationship between the two variables. Testosterone in the HV training week is likely linked to a higher body fat percentage.

Moderately negative correlations within Leptin and EB in the HV training week (r(11) = −0.59, *p* = 0.033), leptin and fat intake (g) in the HV training week (r(11) = −0.60, *p* = 0.03), T in the HV training week and CHO intake (r(11) = −0.66, *p* = 0.006), cortisol HV training week and DXA BFP (r(11) = −0.602, *p* = 0.025), and IL6 and fat intake during LV week (r(11) = −0.56, *p* = 0.048) were found, suggesting that leptin in the HV training week is linked to decreased EB (kcals) and reduced fat intake (g), while T in the HV training week was accompanied by decreased CHO kcal/kg FFM intake. Cortisol in the HV training week is linked to a decrease in body fat percentage, and IL6 in the LV training week tends to decrease with fat intake (g).

Simple linear regressions were calculated to predict hormone levels based on EA, CA, and other variables (i.e., RMR ratio, EI, EEE, DXA BFP, macronutrients, and distance). Significant regression equations were found between DXA BFP and T, leptin (both weeks) cortisol, T and CHO intake, leptin and fat intake, leptin and RMR ratio, and IL6 and EI. Examining T in the HV Week based on DEXA BFP (F(1,11) = 4.91, *p* = 0.049) produced an R^2^ of 0.246. The participants’ predicted T during HV training equals 11.128 + 0.001 (DXA BFP) percentage. The participants’ average T value increased by 0.001 ng/dL for each percentage of body fat. Observing leptin levels in the HV Week based on DEXA BFP results elicited a significant effect (F(1,11) = 40.56, *p* < 0.001), with R^2^ of 0.767. The participants’ predicted leptin levels during HV training equaled 9.15 + 5.18 (DXA BFP) percentage. The participants’ average leptin values increased by 5.18 ng/mL for each percentage of body fat. Leptin levels in the LV Week based on DEXA BFP elicited (F(1,11) = 74.67, *p* < 0.001) an R^2^ of 0.86. The participants’ predicted leptin levels during LV training equal 9.69 + 5.56 (DXA BFP) percentage. The participants’ average leptin values increased by 5.56 ng/mL for each percentage of body fat. Cortisol levels in the HV Week based on DEXA body fat percentage were found (F(1,11) = 6.69, *p* = 0.025), with R^2^ of 0.32. The participants predicted cortisol levels during HV training to be equal to 23.41–0.78 (DXA BFP). The participants’ average cortisol values decreased by 0.78 ug/dL for each percentage of body fat.

A significant regression equation was found between hormones, macronutrients, and caloric intake. During HV week, T levels based on CHO average intake produced a significant effect (F(1,11) = 6.59, *p* = 0.026), with R^2^ of 0.32. The participants’ predicted T levels during HV training were equal to 454.44–0.06 (CHO) grams. On average, participants’ T values decreased by 0.06 ng/dL for each gram of CHO ingested. Leptin levels in the HV training week based on fat intake (F(1,11) = 6.18, *p* = 0.03), with R^2^ of 0.30. The participants’ predicted leptin levels during HV training were 159.7 + −56.17 (fat) kcal. The participants’ average leptin values decreased by 56.17 ng/mL for each kcal of fat ingested. Leptin levels in the LV week, based on the RMR ratio, produced a significant effect (F(1,11) = 7.01, *p* = 0.02) with an R^2^ of 0.34. The participants’ predicted leptin levels during LV training are equal to 1.14–0.26 (RMR ratio). On average, participants’ leptin values decreased by 0.26 of the RMR ratio. When examining IL6 levels in the LV training week based on average caloric intake (EI), a significant regression equation was found (F(1,11) = 5.32, *p* = 0.04), with R^2^ of 0.27. The participants’ predicted IL6 levels during LV training were 3895.61–1728.9 (calorie) kcal. The participants’ average IL6 values decreased by 1728.9 pg/mL for each caloric kcal ingested.

## 4. Discussion

This study examined the effect of EA and CHO availability on reproductive (T and LH) and metabolic (insulin, leptin, cortisol, and IL6) hormones in male endurance-trained athletes. We hypothesized that male endurance-trained athletes who displayed LEA and LCA would present with decreased T, LH, insulin, and leptin, while cortisol and IL-6 would be increased. A secondary purpose aimed to examine secondary measures, including EI, EEE, DXA BFP, and distance milage and their relationships to hormones. We hypothesized that some secondary measures would demonstrate negative relationships with hormones.

Overall, our results partially supported our hypothesis that LEA (≤25 kcal/kg FFM/day) and LCA (<6 g/kg) would be significantly associated with multiple hormones. LEA was associated with increased T and decreased leptin for both training volume weeks and decreased IL6 within the low-volume training week. LCA (<6 g/kg) was linked with increased T, decreased leptin during both training weeks, and increased insulin during the low-volume training week. Our results demonstrated some congruencies with previous laboratory studies that examined males with set LEA states, resulting in decreased insulin [1,15] and leptin [1,15,17,42] while the T levels were not disrupted. Our LEA results (~39–46%) are congruent with most LEA studies for male endurance athletes that have documented a prevalence between 15–76.9% [13,16,43,44,45,46]. Regarding LCA, McGuire et al. [16] found that 92% of males were below the recommended intake of 7–12 g CHO/kg/day. While we used 6 g/kg/day, this is similar to our results (~54–70%) during the two training weeks. Other intervention studies found ~25–60% reduced CHO when reducing LEA. [13,15,42,47]. Lodge et al. [48] found ~85% of female collegiate runners and ~72% of young and elite athletes under-consumed CHO (<6 g/kg).

Hormones: Both weeks demonstrated larger T values than the “normal” range, which was incongruent with the literature examining endurance runners. Previous research has demonstrated that endurance training has a negative effect on T levels in males. [1,4,10,11,12,13,44,45,49] The mechanistic nature of this decrease is currently unknown. The mechanistic nature of the increase compared to decreases in literature is also unknown. One impression of this increase in T may be a small hormonal reaction not seen before, as many previous studies have participants at a much lower LEA than this study’s participants and significantly increased EEE [1,10,11,12,13,15,19,23]. This increase may show the robustness of T and a potential increase rise in T as a response to mild increased stressors of the body when coupled with other variables (LCA, Low leptin, low IL6, higher cortisol, and moderate mileage). There are a few proposed mechanisms for decreases in T. De Souza and colleagues [50] established a “training volume threshold” (~100 km/week), which demonstrated significant negative changes in the male reproductive function [50]. The high volume of endurance running (>104 km/week) showed associations with changes in both sex hormone profiles (decreased T) and quality of semen (decreased mobility and increases in immature cell numbers) [49,50]. Our participants were close to this distance in the HV week (~103 km) but demonstrated high testosterone levels. As this study was observational in nature, energy deficiency (EA~25–29 kcal/kg FFM·d^−1^) and body fat (~13.5%) may not have been low enough, in conjunction with EEE (~670 kcals) and mileage (HV: ~103 km; LV: 58.9 km) may not have been high enough to elicit decreases in T levels. Most participants were resistance-trained and participated in resistance training; the associated literature confirms acutely increased testosterone [51].

While research has shown changes in T levels due to high training loads, LH is not significantly impacted during increases in training loads. Our study did not demonstrate compromised LH levels. Conversely, Kuoppasalm et al. [52] demonstrated with long-term, high-intensity running, roughly 30 min after long-term runs, plasma LH significantly dropped below baseline levels by 42% (moderate run) and 45% (intense run), suggesting intensity is important regarding negative LH outcomes. MacConnie et al. [53] examined highly trained male marathon runners (125–200 km/week) and found the runners had diminished frequency of spontaneous LH pulses, and the amplitude of their LH pulses was decreased compared to healthy controls [53]. These findings suggest that highly trained male athletes may experience a deficiency in hypothalamic gonadotropin-releasing hormones, potentially triggered by sudden increases in EEE and marked reductions in EI and body fat. In contrast to normative values, our examination of metabolic hormone responses found that insulin levels remained within normal limits for males. This is not congruent with previous literature, which demonstrated decreases in insulin due to high EEE and low EI [19,23]. Koehler et al. [15] also found decreases in insulin in relation to EA suppression (15 kcal/kg FFM·d) in male cyclists. Our EA levels, however, may not have been low enough to elicit a negative response in insulin. Further research is needed to understand the mechanism of decreased insulin in LEA and LCA states [54].

Our results found up to ~54% of participants exhibited low leptin levels alongside low DXA BFP (13.55 ± 3.63), which is congruent with Hagmar et al. [20], who examined 18 Olympic male athletes in leanness sports and reported low leptin levels (1.04 ng/mL) as well as low DXA BFP (11.7 ± 3.4%) compared to non-lean sports. Similarly, Leal-Cerro et al. [55] found that marathon runners elicited lower leptin levels than nonobese controls. Typically, body weight/body fat has an analogous relationship with leptin (i.e., weight loss reduces leptin while weight gain increases leptin levels) [56]. It is hypothesized that the reduction of leptin is an acute metabolic signal of starvation and energy conservation [56,57]. The reductions in leptin are important due to the association with the suppression of key endocrine axes (reproductive, growth hormone, IGF-I, and thyroid axes) [57].

Cortisol results were not congruent with previous literature, which saw increases in cortisol due to decreased EI and increased EEE [17,19,58]. Hill et al. [58] and Öniz et al. [17] demonstrated moderate to high-intensity exercise invokes an increase in circulating cortisol levels. Cortisol levels may increase due to an increased need to catabolize other energy sources besides fat stores or a reduction in clearance [19]. Our results did not demonstrate an increase or decrease in cortisol, which may allude to the intensity not being elevated enough or an insufficient decrease of EI and LEA levels to increase cortisol levels. Our results did not demonstrate an increase in IL-6 levels compared to normative data (≤1.8 pg/mL). This is not congruent with previous literature that found increases in IL-6 levels after strenuous exercise as an inflammatory marker [59,60]. However, research reports that a change in IL-6 is an acute response, while our blood draws were done after 24 h of rest from exercise on the 8th day of the study. Fischer [61] examined IL-6 responses to acute bouts and training loads and found a training effect for the downregulation of IL-6. Therefore, the low plasma levels of IL-6 can be characterized as a training adaptation [61]. In conjunction with 24 h rest, this training effect may explain our lower levels of IL-6. While this study was designed as a free-living study, the timing of hormone releases needs to be accounted for in the future for more accurate responses.

A secondary purpose of this study was to examine secondary measures and their relationships/associations with the different hormones. We hypothesized these secondary measures would demonstrate negative relationships with the hormones. We calculated prediction equations from DXA BFP, RMR ratio, macronutrients (CHO and fat), and EI that demonstrated significant regression equations to predict T levels, leptin, cortisol, and IL6 in male endurance-trained athletes. Linear regressions demonstrated DXA BFP was a good predictor for T, leptin [15], and cortisol levels. RMR ratio predicted leptin, which aligns with Stenqvist et al. (2021) [33], who demonstrated that RMR ratios (<0.90) elicited differences in hormone values (decreased T and increased cortisol) compared to those with RMR ratios above (>0.90) in Norwegian male Olympic-level athletes. RMR ratio has also been previously linked to predicting LEA and deficient energy [62]. CHO intake was a good predictor for T, fat intake predicted leptin, and EI was a good predictor for Il6. While these novel prediction equations were statistically significant, they need to be validated to be impactful for clinicians who do not have access to tools for blood testing capabilities and measuring LEA. The ability to use prediction equations to assess T, cortisol, and leptin levels via DXA BFP, LEA with RMR ratios, and nutrition intake could aid in the overall health of male athletes in the future.

### Limitations and Future Directions

There were limitations identified in this study. First, while this study did reach power, the subject count was low (*n* = 13), however, consistent with similar literature examining LEA. Secondly, large standard deviations were found within our measures, partly due to inter-individual variability between participants’ training status, even though all met the inclusion criteria. Another limitation was that EI was based on a 7-day self-reported dietary log, which, while most were valid and reliable, still allowed for subject interference with accurate measures [34]. Double-labeled water would be a more valid and reliable examination of EI. Some measures may have benefited from different measurement styles, including (1) LH, as it is pulsatile and should be measured over 24 h; (2) T would have been more expressive if measured daily; and (3) IL-6, which has demonstrated more acute responses to exercise and may have given more information to inflammatory responses if measured within a few hours after exercise compared to after 24 h of rest at the end of the week. Future studies should examine hormones to (1) identify hormone markers specific to male athletes, specifically T and LH, and (2) more closely to their measurement needs. Future studies should also validate predictive equations for reproductive and metabolic hormones so that clinicians and athletes can assess hormones without blood draws. More intervention studies examining specific EA levels and the response of reproductive and metabolic hormones specific to set EA levels would be beneficial.

## 5. Conclusions

In conclusion, male endurance-trained athletes exhibiting LEA and LCA experienced associated negative hormonal responses. Key variables such as LEA, LCA, DXA BFP, and EI) were significantly related to T, leptin, and cortisol levels, highlighting the physiological impact of LEA, LCA, EI, EEE, and body composition on hormonal processes. These findings are critical for athletes, coaches, and clinicians seeking to optimize the health and performance of male athletes, mainly those prone to LEA, LCA, reduced EI, BFP, and heightened EEE. Based on this study, male distance athletes should monitor their EEE and EI to maintain appropriate levels of EA (≥25 kcal/kg FFM·d-1), CA (>6 g CHO/kg), and DXA BFP (>13.55%) as these levels potentially alter leptin levels. Developing valid and reliable predictive equations for hormones (T, leptin, and cortisol) may become valuable and accessible tools for clinicians and healthcare providers.

## Figures and Tables

**Table 1:** Basic Demographics and Inclusion Criterion for Endurance Trained Male Athletes (*n* = 13). Values are presented in mean (M) ± standard deviation (SD) or number (N) and percentage (%).

Basic Demographics	ALL	
	M	SD
Age (years)	26.08	4.27
Height (cm)	179.91	4.19
Weight (kg)	70.86	6.53
BMI (kg/m^2^)	22.02	1.77
**Ethnicity**	**N**	**%**
African American	2	15.4
Caucasian	10	76.9
Middle Eastern/Kurd	1	7.7
**Education Level**	**N**	**%**
High School Diploma/GED	1	7.7
Attained some College	4	30.8
Bachelor’s Degree	3	23.1
Master’s Degree	4	30.8
Clinical Doctorate	1	7.7
**Inclusion Criterion**	**M**	**SD**
VO_2max_ (mL/kg/min)	62.74	6.96
Free Fat Mass (kg)	65.88	5.60
BIA BFP (%)	6.97	2.19
DXA BFP (%)	13.39	3.56
RMR (kcal)	1785.4	568.8
RMR Ratio	.96	.248

Note: BFP—body fat percentage, BIA—bioelectrical impedance analysis, DXA—dual-energy X-ray absorptiometry, RMR—resting metabolic rate.

**Table 2:** Energy Needs of Male Endurance Athletes *n* = 13. Values are presented in mean (M) ± standard deviation (SD) and *p*-value of paired *t*-tests between High- and Low-Volume weeks.

Energy Intake	HV M ± SD	LV M ± SD	*p*-Value
Calories (kcal)	2724. 7 ± 903.4	2919.9 ± 912.7	0.16
Protein (g)	126.5 ± 44.7	134.5 ± 50.6	0.34
CHO (g)	363.0 ± 130.3	346.3 ± 124.0	0.52
CHO (g/kg)	5.5 ± 1.8	5.3 ± 1.8	0.58
FAT (kcal)	113.7 ± 57.0	105.5 ± 40.1	0.43
**Energy Expenditure**			
EEE (kcal)	897.7 ± 601.1	669.4 ± 480.6	0.01
TDEE (kcal)	3117.2 ± 619.2	2920.1 ± 496.3	0.03
**Training Volume**			
Training Volume (minutes)	409.9 ± 325.0	238.6 ± 155.7	<.001
Distance (milage in km)	103.2± 166.4	58.9 ± 75.3	0.08
**Energy Calculations**			
EA (kcal/kg FFM)	25.7 ± 13.4	30.8 ± 11.1	0.13
EB (kcal)	−392.5 ± 863.0	−249.7 ± 917.7	0.27

Note: CHO—carbohydrate, EEE—exercise energy expenditure, TDEE—total daily energy expenditure, EA—energy availability, EB—energy balance.

**Table 3:** Hormonal Profile Male Endurance Athletes *n* = 13. Values are presented in mean (M) ± standard deviation (SD) and *p*-value of paired t-tests between High- and Low-Volume weeks.

Hormones	HV M ± SD	LV M ± SD	*p*-Value
Testosterone (ng/dL)	1652.11 ±1441.30	1909. 99 ± 1903.96	0.17
LH (mlU/L)	785.77 ± 393.64	841.63 ± 234.65	0.44
Insulin (ulU/mL)	7.12 ± 1.07	8.29 ± 3.63	0.27
Leptin (ng/mL)	0.82 ± 0.61	0.67 ± 0.60	0.13
Cortisol (ug/dL)	12.72 ± 2.79	13.50 ± 2.78	0.25
IL6 (pg/mL)	0.83 ± 0.82	0.56 ± 0.30	0.34

Note: LH—luteinizing hormone, IL6—interleukin-6.

**Table 4:** Compromised Hormones in Relation to LEA and LCA in Male Endurance Athletes *n* = 13. Values are presented in mean (M) ± standard deviation (SD), Chi-Square, and *p*-values between High- and Low-Volume weeks.

LEA High Volume Training Week	N, Percentage	Chi-Square	*p*-Value
Low Leptin	23.1%, *n* = 3	χ^2^ (2) = 4.55	0.10
Low IL6	46.2%, *n* = 6		
High T	30.8%, *n* = 4	χ^2^ (2) = 2.30	0.32
High LH	46.2%, *n* = 6		
**LCA High-Volume Training Week**			
Low Leptin	30.8%, *n* = 4	χ^2^ (1) = 3.34	0.07
Low IL6	53.8%, *n* = 7		
High T	30.8%, *n* = 4	χ^2^ (1) = 0.07	0.80
High LH	53.8%, *n* = 7		
**LEA Low-Volume Training Week**			
Low Leptin	15.4%, *n* = 2	χ^2^ (2) = 3.01	0.22
High T	30.8%, *n* = 4	χ^2^ (2) = 2.30	0.32
High LH	38.5%, *n* = 5		
**LCA Low-Volume Training Week**			
Low Leptin	53.8%, *n* = 7	χ^2^ (1) = 1.051	0.31
High T	38.5%, *n* = 5	χ^2^ (1) = 0.442	0.51
High Insulin	15.4%, *n* = 2	χ^2^ (1) = 1.051	0.31
High LH	69.2%, *n* = 9		

Note: IL6 and LH did not produce a Chi-Square or *p*-value as there were no multiple levels of hormone distribution; degrees of freedom were 1. LEA—low energy availability, LCA—low carbohydrate availability, T—testosterone, IL6—interleukin-6, LH—luteinizing hormone.

## Data Availability

The data presented in this study are available on request from the corresponding author due to propriety reasons implemented by universities.
